# Association of protein arginine deiminase 4 with the myosin-9 motor complex

**DOI:** 10.1016/j.jbc.2025.110513

**Published:** 2025-07-22

**Authors:** Xiaoxing Wang, Fatemeh Moadab, Farheen Shaikh, Jie An, Cecilia Mustelin, Ethan Le, Sadie Van der Bogaerde, Rayan Najjar, Tomas Mustelin

**Affiliations:** Division of Rheumatology, Department of Medicine, University of Washington, Seattle, Washington, USA

**Keywords:** rheumatoid arthritis, PAD4, citrullination, myosin-9, grancalcin, autoantibodies

## Abstract

Genetic association data, immunohistochemistry, and functional experiments implicate protein arginine deiminase 4 (PAD4) in the pathogenesis of rheumatoid arthritis (RA). This disease is characterized by immunity against epitopes with deiminated arginine (=citrulline) originating from a multitude of intra- and extracellular proteins that are modified in this manner only in patients with RA, not in healthy individuals. However, it remains uncertain how, where, and why PAD4 citrullinates these proteins in patients with RA. To gain insights into the physical interactions of PAD4 with other cellular proteins, we identified candidate PAD4-associated proteins by mass spectrometry. PAD4 in neutrophils from patients with RA and healthy controls co-immunoprecipitated with myosin-9 and 20 other proteins, many of which were also present in myosin-9 immunoprecipitates. By immunofluorescence microscopy, PAD4 co-localized with myosin-9, myosin light chain 6, and other associated proteins in RA neutrophils. This was confirmed by proximity ligation assays in intact neutrophils. Inhibition of the motor domain of myosin-9 by blebbistatin resulted in a more diffuse PAD4 location, indicating that myosin-9 serves to transport PAD4 within the cells. However, PAD4 translocation to the nucleus involved dissociation from myosin-9. In complex with PAD4, myosin-9 was citrullinated at both N-terminal and C-terminal sites in patients with RA but not in healthy controls. Citrullinated peptides corresponding to these sites were recognized by IgG autoantibodies in patients with RA. We conclude that at least a portion of intracellular PAD4 in neutrophils interacts physically and catalytically with a myosin-9-containing macromolecular machinery involved in cell migration and transport of organelles and membranes.

The deimination of arginine residues in proteins, also known as protein citrullination, is an irreversible post-translational modification utilized in different physiological processes, such as the regulation of transcription factors involved in stem cell differentiation (1) and counteracting histone methylation by the citrullination of methylated arginines (2, 3, 4). Quantitatively and qualitatively abnormal citrullination is also a hallmark of rheumatoid arthritis (RA), where this modification of Arg residues creates novel sequences that the immune system may recognize as foreign antigens (5, 6, 7). Autoantibodies against citrullinated proteins are present in 70 to 80% of patients with RA(8) but not in other autoimmune or rheumatic diseases (9, 10), and together with the presence of rheumatoid factor, they form the basis for the diagnosis of “seropositive” RA (11).

Of the five human genes encoding for protein arginine deiminases (PADs), PAD2 and PAD4 appear to be responsible for the abnormal citrullination in RA (12, 13, 14, 15, 16). They are present in joint tissue (17) and synovial fluid (18, 19) and they are expressed in immune cells, particularly in neutrophils, which are abundant in the synovial fluid of patients newly diagnosed with RA (20).

An enigma in PAD4 biology stems from its requirement for millimolar concentrations of Ca^2+^ for catalytic activity (21), yet there is evidence that it can citrullinate proteins in the nucleus (1, 22) or cytosol (23, 24) of intact cells where Ca^2+^ concentrations are less than 150 nM, which is 3 to 4 orders of magnitude lower than the enzyme requires *in vitro*. Even potent neutrophil activation accompanied by calcium mobilization only reaches a maximum of 500 nM intracellular Ca^2+^, which is still three orders of magnitudes too low for PAD4 activation *in vitro*. One possibility is that a PAD4-associated protein somehow lowers the requirement for Ca^2+^ (25) and/or provides calcium ions to the enzyme. On the other hand, pores created by polymerized perforin in the membrane of neutrophils by cytotoxic lymphocytes allow for a much greater Ca^2+^ influx and cause a strong activation of intracellular PAD4 and the citrullination of over 30 cellular proteins (26, 27). Many freshly isolated neutrophils from patients with RA already have these poly-perforin pores as well as intracellular citrullination (28). Most of the proteins that are citrullinated in these neutrophils (28) are also found in their citrullinated form in the synovial fluid of patients with RA (29), further supporting the notion that this mechanism of inducing citrullination occurs *in vivo* in patients with RA.

To gain insights into the regulation and location of PAD4, we set out to identify PAD4-associated proteins by a combination of mass spectrometry, co-immunoprecipitation, and immunofluorescence microscopy. We report that PAD4 is associated with a large intracellular motility machinery centered around myosin-9, as well as proteins that may not bind PAD4 *via* myosin-9. We also find that an inhibitor of the motor domain of myosin-9 alters the intracellular distribution of PAD4, suggesting that the myosin-9 machinery controls the location of PAD4 within neutrophils.

## Results

### Mass spectrometry analysis of proteins co-immunoprecipitating with PAD4

**Figure 1:**
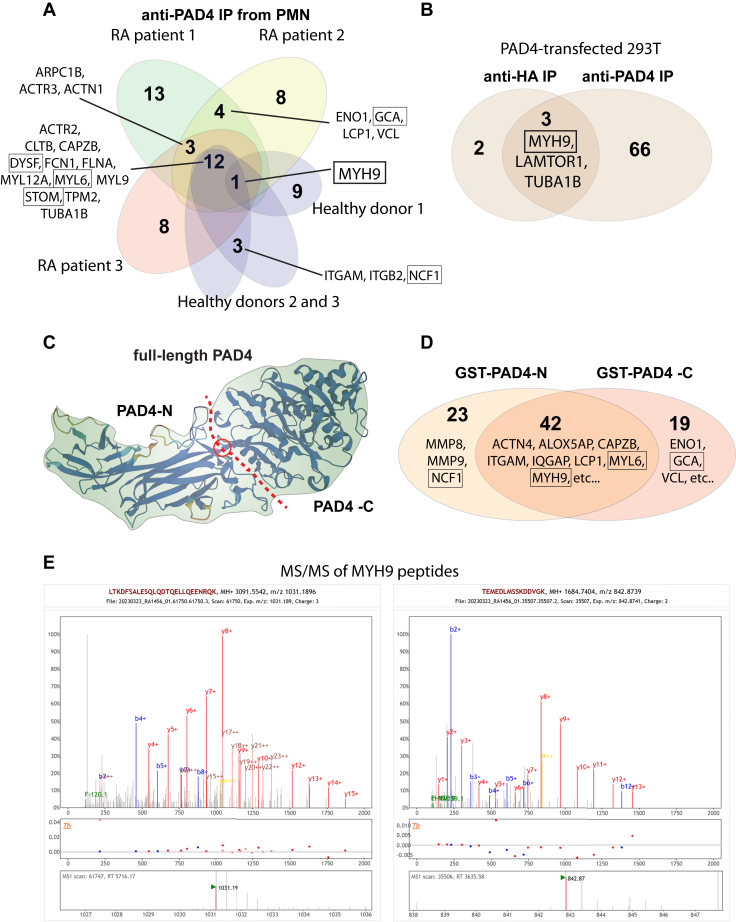
**Summary of mass spectrometry analyses to identify PAD4-associated proteins.***A*, Venn diagram of proteins present in anti-PAD4 immunoprecipitates, but not in control precipitates, from neutrophils obtained from 3 patients with RA and three healthy donors. *B*, Venn diagram of PAD4 immunoprecipitates from transfected 293T cells. *C*, illustration of the hinge (*red circle*) where PAD4 was cut into two halves for the construction of the two GST fusion proteins. *D*, Venn diagram of proteins precipitated by the N- and C-terminal halves of PAD4 fused to GST. *E*, two representative mass spectra of myosin-9 peptides from the immunoprecipitates in *panel A*.

To identify proteins potentially interacting with PAD4, we first used a mouse monoclonal antibody clone OTI4H5 (Abcam) to immunoprecipitate PAD4 from lysates of neutrophils from three different patients with RA and one healthy donor and then subjected the precipitated proteins to LC-MS/MS mass spectrometry using an Orbitrap instrument. Only proteins unambiguously identified by matching peptides (probability = 1 and q-value for matching expected peptides <10^−4^) were considered and all proteins identified in a control precipitate prepared from the same RA neutrophil lysates without the anti-PAD4 antibody were subtracted. The presence of PAD4 itself in the anti-PAD4 immunoprecipitates was validated by unique peptides covering 47.7% of its sequence. In addition, 16 other proteins were present in immunoprecipitates from all 3 patients with RA (Fig. 1*A*). Anti-PAD4 immunoprecipitates from one healthy donor contained only myosin-9 (MYH9), while immunoprecipitates from two additional healthy controls contained most of the same proteins as immunoprecipitates from patients with RA. Additionally, four proteins (ENO1, GCA, LCP1, and VCL) were present in two of the three patients or one patient and the healthy controls. Very similar results were obtained with a polyclonal anti-PAD4 antibody (Abcam) and a human IgG4 antibody against PAD4 (kindly provided by AstraZeneca). Representative mass spectra are shown in Supplemental Fig. S1-S3.

Most of these co-immunoprecipitating proteins are cytoskeletal proteins associated with the myosin-9 machinery for the directional mobility of surface proteins and intracellular organelles, including myosin light chains 6 (MYL6) and 12A (MYL12A), actin-related proteins 2 and 3 (ACTR2, ACTR3), and proteins regulating actin and myosin functions, such as filamin (FLNA), F-actin-capping protein subunit beta (CAPZB), tropomyosin (TPM2 and 3), alpha-actinin-1 (ACTN1), and plastin-2 (LCP1), which bundles actin filaments in a calcium-dependent manner. Tubulin (TUBA1B) and vinculin (VCL) are also cytoskeletal proteins. Grancalcin (GCA) is an abundant Ca^2+^-binding protein in neutrophils. Dysferlin (DYSF) is a protein that translocates in a Ca^2+^-dependent manner to sites of the plasma membrane damage, while stomatin (STOM) is a transmembrane protein that regulates ion channels and neutrophil degranulation. Enolase-1 (ENO1) is a metabolic enzyme and a well-known target for citrullination in RA (30). As a positive control, neutrophil cytosolic factor-1 (NCF1) was also detected in PAD4 immunoprecipitates from two healthy donors, as reported before (31). Peptides corresponding to NCF1 were also present in immunoprecipitates from patients with RA but did not meet our stringent quality criteria (see Methods).

### Immunoprecipitation of PAD4 from transfected non-hematopoietic cells

To test if PAD4 interaction with myosin-9 depended on any neutrophil-specific proteins, we transfected 293T cells (which express myosin-9, but not PAD4) with or without a hemagglutinin epitope (HA)-tagged PAD4 plasmid and immunoprecipitated PAD4 either with anti-PAD4 antibodies or an anti-HA mAb (12CA5). LC-MS/MS of these preparations detected myosin-9 with three peptides in the anti-PAD4 immunoprecipitates and one peptide in anti-HA immunoprecipitates. Tubulin was also present, but no other proteins were seen in PAD4 immunoprecipitates from neutrophils (Fig. 1*B*). As a validation, 40 to 50 distinct peptides from PAD4 itself were present (q ≤ 10^−4^). These findings indicate that the PAD4 interaction with myosin-9 does not depend on neutrophil-specific proteins.

### Proteins bind to the N- or C-terminal half of PAD4

Next, we expressed and purified two glutathione S-transferase-fusion proteins containing the N- or C-terminal halves of PAD4, termed GST-PAD4-N and GST-PAD4-C. These two constructs were based on the crystal structure of PAD4 (32), which shows that PAD4 consists of two separate but closely adjacent domains with a hinge between them (Fig. 1*C*). When these two proteins, as well as GST alone, were mixed with RA neutrophil lysates and then washed and subjected to mass spectrometry, it was evident that 10 of the proteins that immunoprecipitated with PAD4 were also present among the 42 proteins in both samples but not with GST alone (Fig. 1*D*). In addition, the C-terminal half of PAD4 bound α-enolase (ENO1), grancalcin (GCA), and vinculin (VCL), while the N-terminal half bound neutrophil cytosolic factor 1 (NCF1), which is known to associate with PAD4 and to be citrullinated by it (31).

### Mass spectrometry of proteins co-immunoprecipitating with myosin-9

Because myosin-9 was found in every anti-PAD4 immunoprecipitate and was often represented by more peptides than other proteins (even PAD4), we used an anti-myosin-9 antibody to immunoprecipitate it from neutrophils. LC-MS/MS revealed that eight of the 15 proteins that co-immunoprecipitated with PAD4 from neutrophils (CAPZB, DYSF, FLNA, MYL12A, MYL6, STOM, TPM2, TUBA1B), as well as PAD4 and myosin-9 itself, were present (data not shown). Myosin-9 was represented by 101 to 179 distinct peptides (Fig. 1*E*) and PAD4 by 28 distinct peptides. These results support the notion that PAD4 is interacting with a larger myosin-9 protein complex that includes most of the proteins in Figure 1*A*.

### Reciprocal co-immunoprecipitation of candidate proteins and PAD4

**Figure 2:**
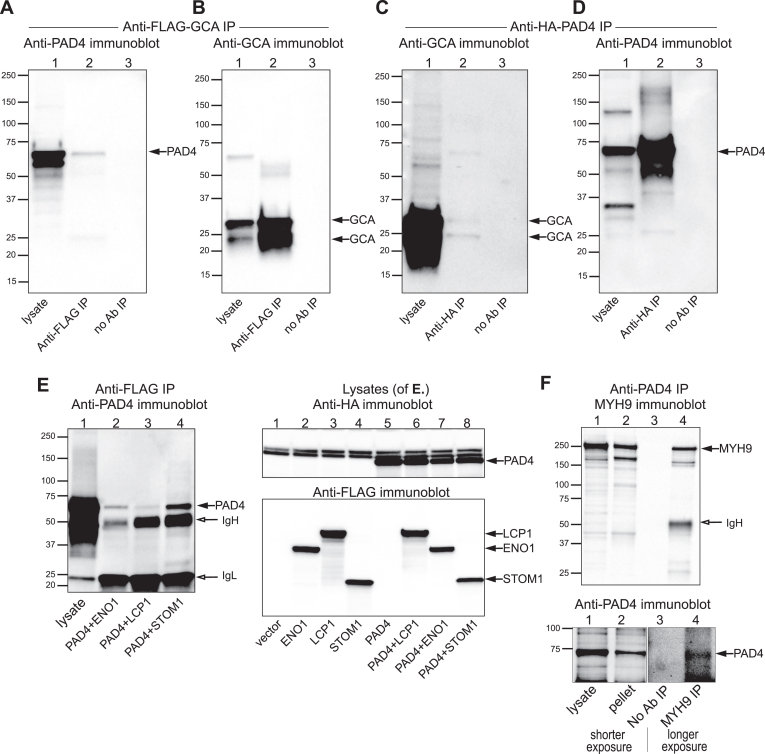
**Co-immunoprecipitation of enolase, plastin-2, stomatin, and myosin-9 with PAD4 from co-transfected 293T cells or from THP-1 cells.***A*, anti-PAD4 immunoblot of total lysates, anti-FLAG immunoprecipitates, or mock immunoprecipitate from 293T cells transfected with HA-tagged PAD4 and FLAG-tagged GCA. *B*, anti-GCA immunoblot of the same samples as in panel A. *C*, anti-GCA immunoblot of total lysates, anti-HA or mock immunoprecipitates from the same transfected 293T cells as in panels A and B. *D*, anti-PAD4 immunoblot of the same samples as in panel C. *E*, *Left* panel, anti-PAD4 immunoblot of total lysates (lane 1) or anti-FLAG immunoprecipitates (lanes 2-4) from 293T cells transfected with HA-tagged PAD4 alone (lane 1) or with FLAG-tagged ENO1, LCP1, or STOM1 (lanes 2-4). *Right panel*, control anti-HA immunoblot (*upper*), and anti-FLAG immunoblot (*lower*) of 293T cells transfected with empty vector (lane 1) or FLAG-tagged ENO1, LCP1, or STOM1 alone (lanes 2-4) or together with HA-tagged PAD4 (lanes 6-8) or HA-tagged PAD4 alone (lane 5), as indicated. *F*, anti-MYH9 immunoblot (*upper panel*) and anti-PAD4 immunoblot (*lower panel*) of THP-1 cell lysates (lane 1), insoluble pellet (lane 2), beads-only mock immunoprecipitate (lane 3), anti-MYH9 immunoprecipitate (lane 4), and isotype control immunoprecipitate (lane 4). The two first lanes in the *lower panel* represent a shorter exposure, lanes three and four are a longer exposure to visualize PAD4.

Next, we used co-immunoprecipitation to validate associations of candidate proteins with PAD4 by immunoblotting. Compared to mass spectrometry, this method is more quantitative but also much less sensitive and dependent on the quality of commercially available antibodies. For this reason, we first co-transfected 293T cells with expression plasmids for HA-tagged PAD4 and FLAG-tagged GCA (Fig. 2, *A*–*D*), followed by immunoprecipitation with anti-HA or anti-FLAG and immunoblotted with the reciprocal antibody, or antibodies against PAD4 or GCA. These experiments showed a clear and specific co-immunoprecipitation, albeit to a relatively modest stoichiometry. Similarly, PAD4 was immunoprecipitated by the and-FLAG mAb from cells co-transfected with HA-tagged PAD4 together with FLAG-tagged ENO1, LCP1, or STOM (Fig. 2*E*). Immunoblots of the lysates confirmed that these proteins were well-expressed (Fig. 2*F*). Finally, endogenous myosin-9 was readily detected in THP-1 monocytic leukemia cell lysates (some also in the insoluble pellet) and in anti-myosin-9 immunoprecipitates (Fig. 2*F*, upper panel), which also contained detectable endogenous PAD4 (Fig. 2*F*, lower panel). In contrast, immunoprecipitates obtained without antibodies or with an isotype control did not contain either protein. An additional experiment with two different anti-PAD4 antibodies is shown in Supplemental Fig. S4*A*.

### Co-localization of PAD4 with myosin-9 and other interactors in cells

**Figure 3:**
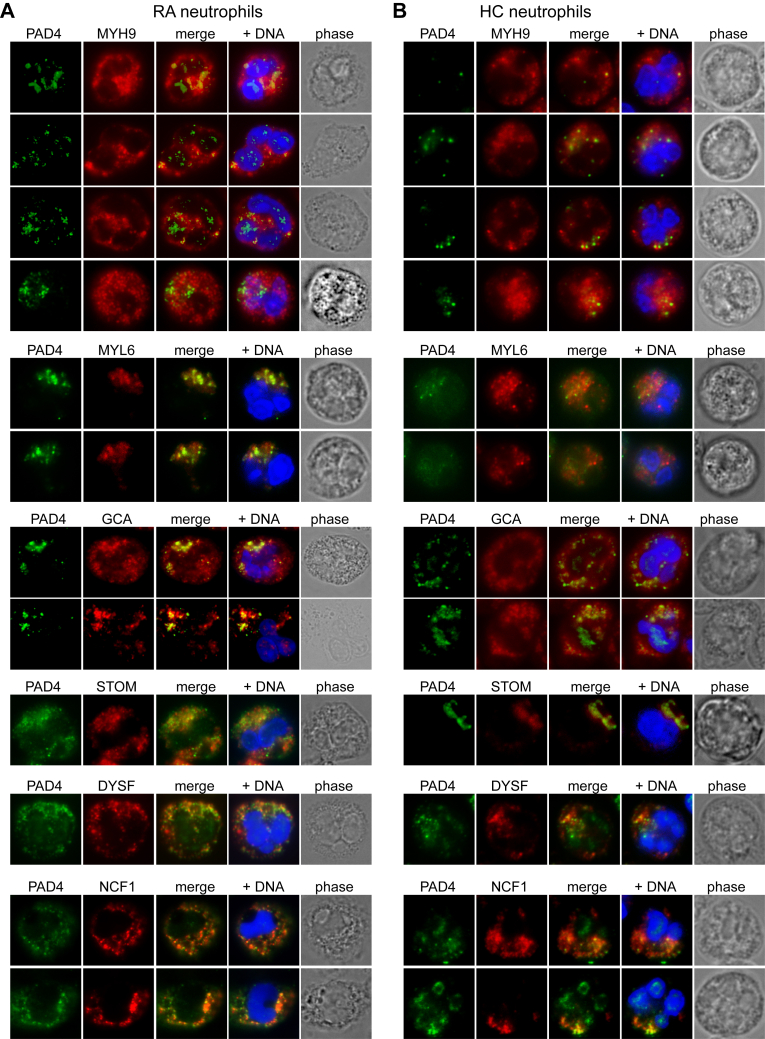
**Immunofluorescence microscopy of PAD4 and putative interactors.***A*, RA neutrophils stained for PAD4 (*green*) and the indicated proteins (*red*): MYH9, MYL6, GCA, STOM, DYSF, and NCF1. DNA is in *blue*, and the last panel in each row is a phase-contrast image of the same cell. Four (MYH9) or two (MYL6, GCA, NCF1) or one (STOM, DYSF) representative neutrophils from nine patients are shown. *B*, healthy control (HC) neutrophils stained with the same antibodies as in A.

**Figure 4:**
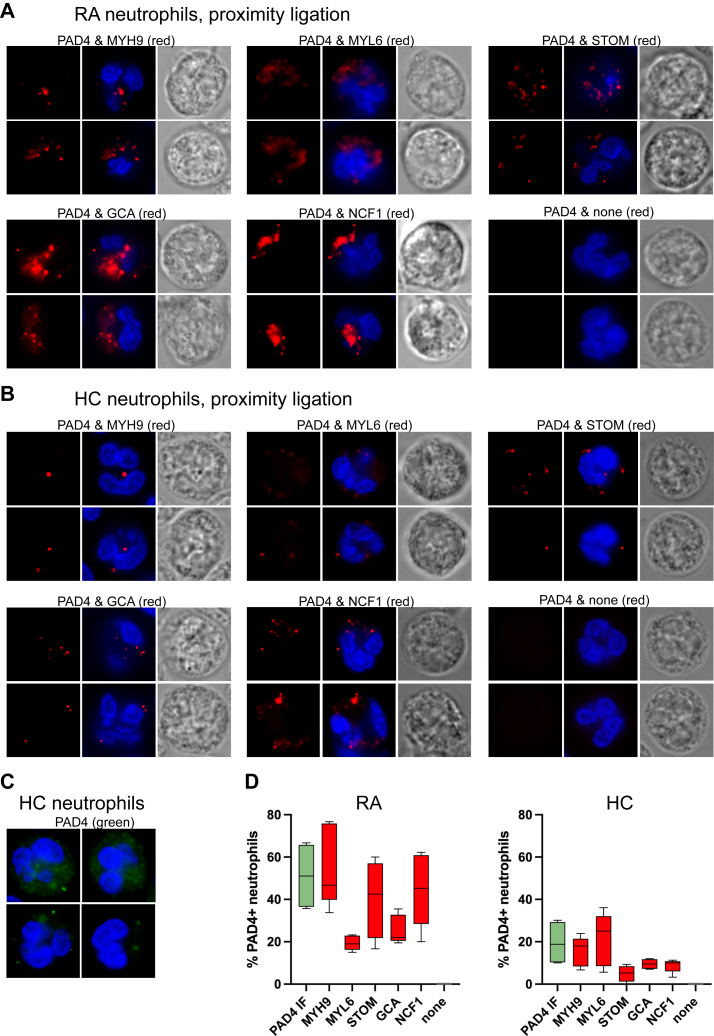
**Close proximity of PAD4 with select interacting proteins.***A*, proximity-ligation assays in RA neutrophils for the pairs of PAD4 plus MYH9, MYL6, STOM, GCA, NCF1, or no second antibody, as indicated. Two representative neutrophils are shown for each pair. *B*, proximity-ligation assays in healthy control (HC) neutrophils with the same antibody pairs. *C*, PAD4 staining of HC neutrophils to illustrate the often weaker staining. *D*, Box plots showing the percent of neutrophils with detectable PAD4 (*green* bar) or positive proximity-ligation signal (*red* bars) counted from 5 to 6 views of 10 to 50 cells each. The boxes denote 10–90th percentile and the bar in the box is the mean.

PAD4 exists in resting neutrophils in a set of cytoplasmic granular structures (31), but assumes a more nuclear localization after treatment of the cells with a calcium ionophore. In RA, the nuclear location is also seen in a subset of freshly isolated neutrophils without any treatment, presumably due to stimuli present in the patient. Staining of neutrophils from patients with RA (Fig. 3*A*) or healthy controls (Fig. 3*B*) for PAD4 and myosin-9 showed a somewhat variable pattern of co-localization: most of the cytosolic PAD4 staining coincided with myosin-9 staining, but myosin-9 was more widely distributed within neutrophils than PAD4. RA neutrophils with nuclear PAD4 did not have any myosin-9 within their nucleus (see also Fig. 4). Neutrophils from healthy controls (Fig. 3*B*) did not have nuclear PAD4 and had weaker cytosolic PAD4 staining, which nevertheless overlapped with myosin-9 as well as MYL6, GCA, STOM, DYSF, and NCF1.

To again test if PAD4 association with myosin-9 depends on other neutrophil proteins, we transfected 293T cells with HA-tagged PAD4 and stained them for PAD4 and myosin-9 or MYL6, showed a similar co-localization (Fig. S4*B*). These data indicate that PAD4-MYH9 interactions can occur in the absence of neutrophil-specific proteins. The negative control, healthy donor neutrophils stained with secondary antibodies alone are shown in Supplemental Fig. S4*C*.

### Close association revealed by proximity ligation assay

Proximity ligation assay (PLA) is a method using antibodies conjugated with complementary DNA oligonucleotides developed to demonstrate that two macromolecules are less than 40 nm apart (33, 34, 35, 36). Using this method, we find that a red fluorescent signal indicating such very close proximity was obtained in neutrophils from patients with RA (Fig. 4*A*) or healthy controls (Fig. 4*B*) for PAD4 in combination with MYH9, MYL6, STOM, GCA, and NCF1 (as a positive control), while a negative control consisting of only anti-PAD4 was entirely devoid of any signal. The observed patterns resembled the yellow pixels in Figure 3, *A* and *B*, but were comparatively stronger for GCA and NCF1 than for the other proteins. The weaker signals in healthy donor neutrophils compared to RA neutrophils paralleled the weaker staining for PAD4 in healthy donors (Fig. 4*C*). To quantify these findings, we counted the percentage of neutrophils with positive PLA signals (Fig. 4*D*). In RA, approximately 50% of neutrophils contain detectable PAD4, while only 20% of healthy neutrophils are positive. These percentages are closely matched by the portion of neutrophils showing a positive PLA signal for MYH9, while the proximity signals for MYL6 and GCA were present in considerably fewer cells in RA, and intermediate for STOM and NCF1. In healthy controls, the proximity signal with MYL6 was similar to MYH9, while it was lower for STOM, GCA, and NCF1. These data indicate a very close proximity of PAD4 and MYH9 rather than just juxtaposition of fluorescence signals obtained in regular immunofluorescence microscopy.

### Effects of myosin inhibitors and calcium ionophore

**Figure 5:**
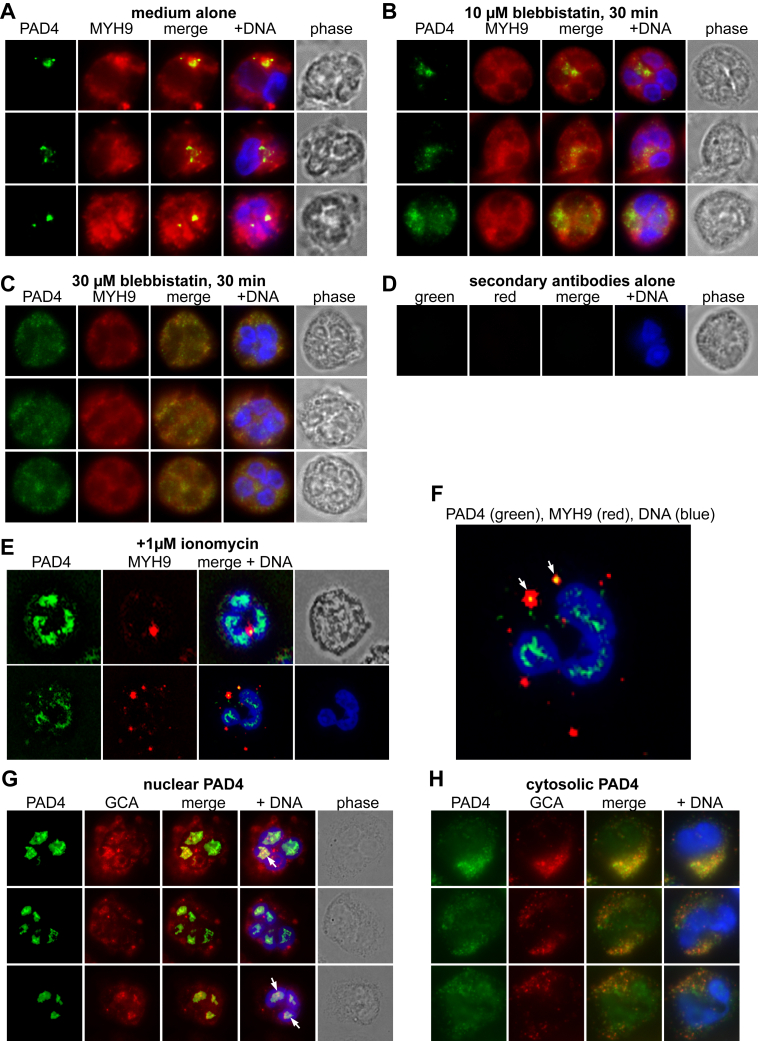
**Effects of myosin-9 inhibition or ionomycin on PAD4 localization.***A*, RA neutrophils stained for PAD4 (*green*) and myosin-9 (*red*) after a 30 min incubation at 37 °C. *B*, same staining of neutrophils incubated with 10 μM blebbistatin for 30 min at 37 °C. *C*, same for neutrophils incubated with 30 μM blebbistatin for 30 min at 37 °C. *D*, negative control staining with secondary antibodies alone. *E*, PAD4 (*green*) and myosin-9 *(red*) in neutrophils treated with 1 μM ionomycin for 30 min at 37 °C. *F*, magnified view of the third panel, second row, in panel E. *G*, RA neutrophils stained for PAD4 (*green*) and grancalcin (*red*) focusing on neutrophils with nuclear PAD4. *H*, same staining of neutrophils with cytosolic PAD4. Note that grancalcin is nuclear only when PAD4 is nuclear.

To test if inhibiting the ATPase activity of myosin-9, which is instrumental for its motility function, would have any impact on the location of PAD4, we treated neutrophils with the type II myosin (including myosin-9 (37)) inhibitor blebbistatin, which blocks myosin II in an actin-detached state with an IC_50_ of 5 μM (38, 39). Compared to untreated controls (Fig. 5*A*), a 30-min treatment with 10 μM blebbistatin led to a somewhat more diffuse staining of myosin-9 and a less concentrated granular PAD4 staining (Fig. 5*B*). At 30 μM of blebbistatin, these effects were more pronounced: both myosin-9 and PAD4 were spread all over the cytosol in very small specks (Fig. 5*C*). Blebbistatin did not impact the viability of the neutrophils but made them even more smoothly spherical than untreated neutrophils (*i.e.*, lacking pseudopods and protrusions). A negative staining control with secondary antibodies alone is shown in Figure 5*D*.

The calcium ionophore ionomycin causes a rapid nuclear translocation of PAD4 (40, 41). PAD4 also has a nuclear location in neutrophils isolated from a subset of patients with RA, perhaps representing those with more activated neutrophils *in vivo*. When neutrophils were treated with ionomycin and stained for PAD4 and myosin-9, it was clear that the two were no longer extensively co-localized: PAD4 was largely nuclear, while myosin-9 had aggregated in larger macromolecular structures, which only contained a small amount of PAD4 (Fig. 5, *E* and *F*).

Unlike the dissociation of PAD4 from myosin-9 during nuclear translocation, GCA accompanied PAD4 into the nucleus (Fig. 5*G*). In cells with only cytosolic PAD4, GCA was also only seen in the cytosol (Fig. 5*H*). We conclude that grancalcin can translocate with PAD4 to (and/or from) the nucleus and, hence, must interact with PAD4 independent of myosin-9.

### Myosin-9 in complex with PAD4 is citrullinated

**Figure 6:**
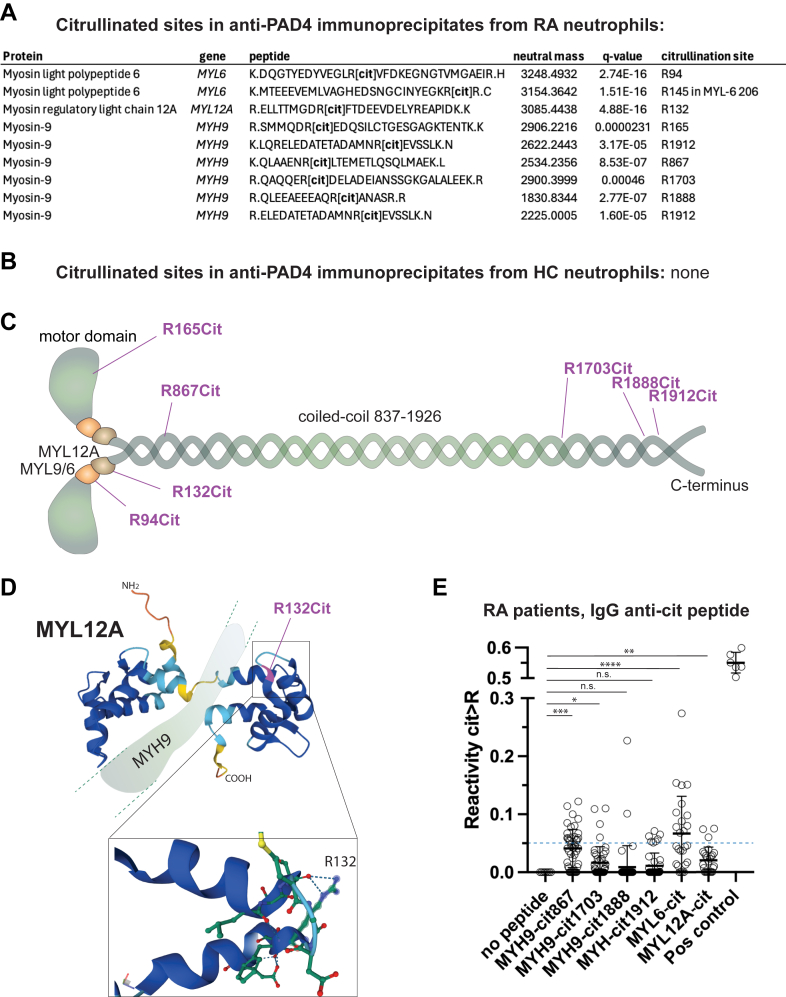
**Citrullination of myosin-9 hexamers in anti-PAD4 immunoprecipitates.***A*, citrullinated peptides in MYL6, MYL12A, and MYH9 in anti-PAD4 immunoprecipitates from patients with RA. *B*, Stating the absence of any detectable citrullination in anti-PAD4 immunoprecipitates from healthy controls (HC). *C*, schematic view of the location of the citrullinated arginine residues in a hexamer of myosin-9 heavy chains with the associated myosin light chains MYL6 and MYL12A. *D*, location of the citrullinated residue R132 in MYL12A to illustrate the loss of a stabilizing interaction with a nearby -OH by the loss of the Arg charge during citrullination. *E*, ELISA for IgG autoantibodies recognizing citrullinated peptides corresponding to the indicated citrullination sites in MYH9, MYL6, and MYL12A. Binding to the same peptides in their unmodified (Arg, R) version was subtracted from all values. 0.05 was designated as a cut-off based on non-specific binding to healthy control IgG. The positive control peptide was a citrullinated peptide corresponding to a putative citrullination site in the HERV-K108 envelope protein.

Myosin-9 has been found in its citrullinated form in the synovial fluid of patients with RA (29) as well as in neutrophils undergoing Ca^2+^-ionophore-induced hypercitrullination (28). The multitude of peptides derived from myosin-9 in our mass spectrometry of PAD4 immunoprecipitates also allowed for an analysis of citrullinated arginine residues. Based on the mass change of deiminated Arg (157.09Da), five citrullinated sites were detected in myosin-9, R165, R867, R1703, R1888, R1912 (Fig. 6*A*). Citrullinated peptides form MYL6 and the regulatory myosin light chain-12A (MYL12A), which form a hexamer with MYH9 (2 of each protein), were also present (Fig. 6*A*). Representative mass spectra are shown in . In contrast, MYH9, MYL6, and MYL12A, present in anti-PAD4 immunoprecipitates from healthy controls, did not contain any citrullinated residues (Fig. 6*B*).

The citrullination sites in myosin-9 were clustered in the N-terminal end in, and near, the motor domain of the myosin-9 protein as well as toward the C-terminal end of its rod-like tail and prior to its phosphorylated C-terminus (Fig. 6*C*). The former are also close to the binding sites for MYL6 and MYL12A next to the motor domain. The citrullination of MYL12A at R132 is predicted to remove a stabilizing connection between two α-helices provided by the side chain of R132 on the C-terminal half of MYL12A (Fig. 6*D*). Besides possibly affecting myosin-9 function and creating potentially immunogenic epitopes for the immune system, these modified residues reveal where PAD4 interacts transiently with myosin-9 during catalysis but do not illuminate where PAD4 binds myosin-9 more stably.

### Sera of patients with RA contain ACPA-binding myosin-9 and light chain citrullinated epitopes

To test if the citrullination of myosin-9 and the myosin light chains result in immunogenic epitopes recognized by ACPA in patients with RA, we screened sera from 30 patients with RA and 20 healthy controls (HC) for IgG autoantibodies against citrullinated peptides corresponding to myosin-9 residues R867, R1703, R1888, and R1912, as well as R94 of MYL6 and R132 of MYL12A. By subtracting reactivity against the corresponding unmodified (Arg) peptides, we considered patient sera with reactivity values > 0 to represent citrulline-specific antibodies. Taking 0.05 as a cut-off for background reactivity (dotted blue line in Fig. 6*E*), RA patient ACPA reacted best with citrullinated MYH9-R867 and MYL6-R94 peptides, while only a few patients reacted with citrullinated MYH9-R1888, MYH9-R1912, or MYL12A-R132 (Fig. 6*E*). These data indicate that at least some of the citrullination sites in MYH9 and MYL6 are immunogenic in the context of RA disease.

## Discussion

Taken together, our results imply that PAD4 in neutrophils from patients with RA and healthy controls are associated with the myosin-9 motor machinery in cytosolic structures, except the nucleus. Blocking the motor function of myosin-9 resulted in a much less focused intracellular distribution of PAD4, indicating that the ATPase of myosin-9 is needed for transporting PAD4 to certain locations or protein complexes. The ATPase motor domain of myosin-9 is a key motility generating component of the myosin-actin cytoskeleton and responsible for the movement and organization of organelles and membrane structures in the neutrophil.

**Figure 7:**
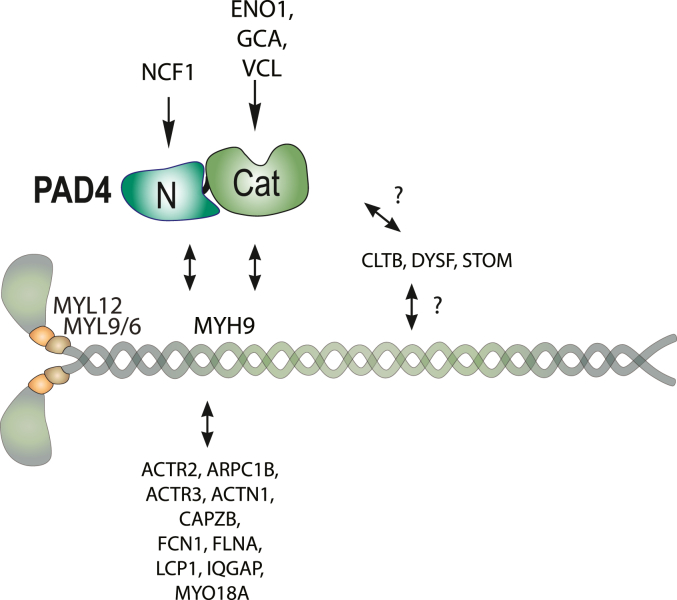
**Schematic representation of PAD4 interactions with myosin-9 and other proteins.** PAD4 is illustrated as two domains as in Fig. 1 and is proposed to interact with NCF1 *via* is N-terminus, with ENO1, GCA, and VCL *via* its C-terminus, and with myosin-9 and its associated proteins (below MYH9) *via* both domains. The binding modes of CLTB, DYSF, and STOM (*right side*) remain unknown.

Because numerous other proteins, such as myosin light chains (at least MYL6, 9, and 12A), actin filaments, actin-binding proteins and regulators, are also present in these macromolecular aggregates, it is difficult to ascertain whether PAD4 binds directly to myosin-9 or to one or several other proteins associated with myosin-9 rather than to myosin-9 itself. These possibilities are not mutually exclusive. However, the proximity ligation assay demonstrates that PAD4 is in very close proximity to myosin-9 and as is MYL6 (hence also MYL12A). The citrullination of myosin-9, MYL6, and MYL12A found in PAD4 immunoprecipitates from patients with RA, but not healthy controls (Fig. 6), also demonstrates that PAD4 interacts physically with all three proteins during citrullination. On the other hand, the translocation of PAD4 to the nucleus without myosin-9 also argues that the interaction is regulated, likely by a Ca^2+^-triggered mechanism. Our data also suggest that grancalcin interacts with PAD4 independently of myosin-9, since grancalcin translocates to the nucleus when PAD4 does. Proximity labeling for PAD4 plus grancalcin was more prominent (Fig. 4*D*) than PAD4 plus myosin-9. We also find it very likely that many of the other cytoskeletal proteins associate with myosin-9 and therefore only indirectly with PAD4 (Fig. 7). The mode of interaction with CLTB, DYSF, LCP1, and STOM remains unclear.

Together with ENO1, GCA was found in association with the C-terminal half of PAD4 (Fig. 1*D*). Grancalcin is particularly interesting because it is an abundant Ca^2+^-binding protein in neutrophils (42) and associates with LCP1 (43). It has been suggested to regulate Ca^2+^-dependent enzymes like calpain and, hence, emerges as a candidate for the PAD4-interacting protein that allows PAD4 to be catalytically active in the nucleus (1, 22), where it appears to accompany PAD4 (Fig. 5*H*), or in the cytosol (23, 24) despite the much too low Ca^2+^ concentration compared to what PAD4 requires *in vitro*. Further studies will be needed to dissect the mode of PAD4–GCA interaction and the role of Ca^2+^ binding to the complex of them.

We previously reported that a portion of PAD4 also exists in a complex with the cytosolic components of the nicotinamide adenine dinucleotide phosphate (NADPH) oxidase complex that catalyzes the oxidative burst in neutrophils, namely NCF1, NCF2, and NCF4 (31). We also detected NCF1 in the present study by mass spectrometry. NCF1 was also present in samples of neutrophil proteins bound to the GST-PAD4-N protein, but not PAD4-C, suggesting that NCF1 associates with the N-terminus of PAD4 (Fig. 7). In addition, the proximity ligation assay (Fig. 4) confirms the close association of PAD4 with NCF1. Furthermore, NCF1 is citrullinated in synovial fluid from patients with RA (29). This is also true for myosin-9, PAD4 itself, and many of the proteins we find in complex with PAD4 (29, 44). Collectively, these findings support the notion that intracellular neutrophil citrullination events contribute to the citrullinome in patients with RA.

An intriguing aspect of our data was not a significant difference in numbers of proteins in anti-PAD4 immunoprecipitates between patients with RAand healthy donors, but rather the citrullination of some of them exclusively in the patients with RA. While the precise set of proteins co-immunoprecipitated with PAD4 varied somewhat from patient to patient, as well as between healthy donors, these differences probably relate to the total amount of PAD4 rather than relevant PAD4 biology. On the other hand, the striking difference in citrullination is likely relevant to the molecular mechanisms of RA: We recently reported that freshly isolated neutrophils from patients with RA, but not healthy donors, have poly-perforin pores on their surface (28) indicating that they are targeted by cytotoxic lymphocytes *in vivo*. These pores allow for a large influx of Ca^2+^ activating PAD4 to citrullinate proteins that are in physical proximity of it, including the proteins identified here. Indeed, proteins citrullinated after ionomycin treatment include myosin-9 and NCF1 (44). Our working hypothesis (45) is that RA entails the generation of pathological citrullination sites distinct from physiological citrullination, rather than loss of tolerance against citrullinated peptides present in healthy individuals. As neutrophils are not targeted by cytotoxic lymphocytes in healthy individuals, we surmise that this targeting and the large influx of Ca^2+^ that poly-perforin pores mediate is pathological and RA-specific. Consistent with this hypothesis, we find that some of the myosin-9 and myosin light chain citrullination sites are recognized by autoantibodies in our RA serum samples.

In conclusion, our findings advance the understanding of PAD4 and its many interactions with regulators and substrates, opening new avenues for further dissection of its physiological and pathophysiological functions.

## Experimental procedures

### Patients with RA

Freshly drawn blood from deidentified patients with RA (n = 30) and healthy controls (n = 20) and serum samples from Patients with RA and healthy controls were from the University of Washington Rheumatology Biorepository with approval of the Institutional Review Board (STUDY00006196). Informed written consent was obtained from all participants according to the Declaration of Helsinki.

### Neutrophil isolation

Neutrophils were isolated from freshly drawn blood by gradient centrifugation on PolymorphPrep (CosmoBio) according to the manufacturer's instructions. The cells were washed and suspended in DMEM high glucose (# 11-965-118, Gibco, ThermoFisher Scientific).

### Antibodies

Anti-PAD4 (Abcam, #ab128086 and Sino Biological, #11072-T16-50), human anti-PAD4 mAb (gift from AstraZeneca), anti-MYH9 (Proteintech, #50-172-9771, ThermoFisher #A304-490A and Invitrogen #PIPA529673), anti-MYL6 (ThermoFisher #PA5-106803, anti-GCA (ThermoFisher #PA5-57124), anti-dysferlin (Invitrogen, # PA5-53546), anti-NCF1/p47phox mAb (Abcam, # ab181090), anti-Plastin1 (Invitrogen, #PA5-852161:100), anti-Stomatin (Proteintech # 12046-1-AP), anti-HA mAb (12CA5 from BioXcell), anti-FLAG (DYKDDDDK) rabbit mAb (D6W5B, Cell Signaling Technology #14793). Normal Rabbit IgG (Millipore Sigma #NI01-100UG) was used as an isotype control antibody. Secondary Antibodies (all from Invitrogen) were goat anti-mouse IgG secondary Ab (AF647 #A-21,235, AF488 #A-11,001), and goat anti-rabbit IgG (AF647 #A-21,245). Non-specific binding of antibodies to FcγRII was prevented by including anti-human CD32 (Thermo Fisher Scientific, #MAB1300SP) and normal goat serum (ThermoFisher, #50062Z).

### Plasmids and transfection

Expression plasmids for full-length PAD4 (NM_012387.3), grancalcin (GCA; NP_001317196), enolase-1 (ENO1; NP_001419.1), plastin-2 (LCP1; NP_002289.2), and stomatin (STOM1; NP_004090.4) were synthesized by Twist Bioscience as eukaryotic expression plasmids with C-terminal HA or N-terminal FLAG epitopes. The plasmids for PAD4 and other proteins were transfected into 293T cells by Lipofectamine 3000 (Thermo Scientific #L3000015) according to manufacturer’s instruction. Cells were harvested 48 h–72 h post transfection.

The N-terminal (aa 1-293) and C-terminal (aa 294-600) halves of PAD4 were synthesized as prokaryotic expression plasmids, pGEX2TK (GenScript), with glutathione S-transferase fused to the N-terminal of truncated PAD4. Plasmid DNA was transformed into *Escherichia coli* BL21 DE3 strain and plated on an LB agar plate with 100 μg/ml of ampicillin. Transformed GST fusion single colonies were inoculated into LB/Amp broth and cultured at 37 °C overnight with shaking. The culture was then inoculated into 50 ml LB/Amp broth at 1:10 and shaken at 37 °C until OD600 reached 0.4 to 0.6. After induction by 0.5 mM IPTG (Fisher Scientific) at 30 °C for 4 h, the GST fusion protein was immobilized onto glutathione agarose beads using the Pierce GST protein interaction pull-down kit (Thermo Scientific) according to the manufacturer’s instruction.

### Immunoprecipitation and mass spectrometry

Transfection 293T cells or neutrophils were lysed by mixing 10^7^ cells in 500 μl of ice-cold lysis buffer (25 mM Tris/HCl, pH 7.4, 150 mM NaCl, 1% TritonX-100, and 1xHalt protease and phosphatase inhibitor cocktail (Thermo Fisher Scientific, #78440)) with rotating at 4 °C for 15 min and clarified by centrifugation at maximum speed for 15 min at 4 °C. Lysates were preincubated on ice for 1 h with anti-mouse IgG-coupled beads (protein G agarose beads, Thermo Fisher Scientific, #20398) with agitation, centrifuged for 5 min, and the supernatant transferred to a new tube. In parallel, 5 μg per sample of anti-PAD4 or anti-HA mAb were incubated with anti-mouse IgG-coupled beads at 4 °C for 30 min, which were then washed once in lysis buffer, mixed with the lysates, and incubated on ice for 1 h. The beads were then washed 5 times in lysis buffer, once in lysis buffer with 500 mM NaCl, once with lysis buffer, and three times with phosphate-buffered saline (PBS). After another three times washes of 20 mM ammonium bicarbonate, on-bead trypsin digestion was performed at 37 °C for 5 h followed by reduction and alkylation. A C18 column (Thermo Fisher Scientific, #89873) was used to purify and concentrate peptides. LC-MS/MS was performed at the University of Washington’s Proteomics Resource on Orbitrap Exploris 480.

To enrich for cellular proteins binding the N- or C-terminal halves of PAD4, beads with GST-PAD4-N or GST-PAD4-C were incubated with RA neutrophil lysates at 4 °C overnight with gentle rocking. After washing for three times with lysis buffer, PBS, and 20 mM ammonium bicarbonate, on-bead trypsin digestion was performed at 37 °C for 4 h followed by reduction, alkylation, and C18 column clean up as described above. The digested peptides were subject to LC-MS/MS as the immunoprecipitates.

Data analysis used the non-redundant protein reference data base and results filtered for probability = 1, and precision of match with predicted peptides (‘Expect value’) q < 10^−4^. Peptides with a predicted C-terminal citrulline are false positives and were discarded. Next all proteins detected in control precipitates obtained with the same procedure, but without antibody, were subtracted from the lists of hits. Finally, immunoprecipitates from 3 separate Patients with RA and one healthy donor were compared and the hits ranked by their presence in two or more samples. The total number of unique non-overlapping peptides in these proteins were also counted.

### Co-immunoprecipitation and immunoblotting

Empty pCMV vector and HA-tagged PAD4 plasmid alone, FLAG-tagged GCA, ENO1, LCP1, or STOM1 either alone or combined with HA tagged PAD4 plasmids were transfected into 293T cells. After 48 h to 72 h, the cells were lysed by Pierce IP Lysis Buffer (Thermo Scientific # 87787). Immunoprecipitation was performed with anti-FLAG resin or anti-HA Agarose (Thermo Scientific #26181) for HA-tagged PAD4. SDS-PAGE was run on a Criterion Cell (Bio-Rad#135BR) 4 to 15% Criterion TGX Precast Gel (Bio-Rad # 5671083). Western Blot was run on the Criterion Blotter (Bio-Rad # 560BR) with Immobilon Transfer Membranes (Millipore#ISEQ00010). Immunoblotting was carried out with rabbit anti-GCA (1:1000), rabbit anti-PAD4 (1:1000), mouse ReadyTag anti-HA (1 μg/ml) for HA-tagged PAD4, anti-FLAG rabbit mAb, and developed with horse radish peroxidase-conjugated anti-rabbit IgG (1:5000, GE Healthcare #NXA934V) or Anti-Mouse IgG (1:5000, GE Healthcare# NXA931V) and Enhanced Chemiluminescence imaging captured by ChemiDoc Imaging System and Image Lab software (Bio-Rad).

THP-1 cells were lysed in Pierce IP Lysis Buffer (Thermo Scientific #87787) supplemented with protease inhibitors and 1 mM EGTA. The lysate was centrifuged at 14,000×*g* for 30 min at 4 °C, and the pellet was saved for further analysis. The supernatant was pre-cleared by incubating with protein G magnetic beads (Thermo Fisher #10-007-D) for 10 min. Equal amounts of clarified lysate were then incubated for 2 h with anti-Myosin 9 (Thermo Fisher #A304-490A) or Normal Rabbit IgG (Millipore Sigma #NI01-100UG), both pre-bound to protein G beads as per the kit (Thermo Fisher #10-007-D) instructions. Antibody was used at 5 μg per mg of protein, and beads alone served as a negative control. Following three washes, the beads were resuspended in 100 μl of SDS-PAGE buffer and boiled for 5 min. A quarter of the sample was analyzed by Western blot, with 1% input loaded for reference.

### Immunofluorescence microscopy

Freshly isolated neutrophils were cultured for 30 min at 37 °C on 96-well glass-bottom plates (Cellvis #P96-1-N) pre-coated with poly-L-lysine (Millipore-Sigma #P4707). Some neutrophils were treated with 10-30 μM Blebbistatin (Cayman Chemical, # NC0527592) or 1 μM Ionomycin (Millipore-Sigma, #10634) for 30 min in serum-free RPMI 1640 (Gibco, # 11-875-093).

The adherent cells were fixed with 4% paraformaldehyde solution for 20 min, followed by two washes with PBS. Cells were permeabilized with 0.1% Triton X-100 in PBS for 10 min followed by three washes with PBS. To prevent nonspecific binding, neutrophils were blocked using 10% normal mouse and/or goat serum in BlockAid solution with anti-human FcγRII antibody overnight at 4 °C. Neutrophils were then incubated with a 1:100 dilution of mouse anti-PAD4 mAb, followed by three washes with PBS. The cells were subsequently stained with goat anti-mouse IgG secondary Ab conjugated with Alexa Fluor 647 (red). Negative controls consisted of cells stained without primary antibodies or with only secondary antibodies. DNA was stained with either DAPI or Hoechst33342. Images were captured at 40×, 60×, or 100× magnification using a Keyence BZ-X800 Fluorescence microscope and analyzed using BZ-X800 Analyzer software.

### Proximity ligation assay

Neutrophils were cultured, fixed, and permeabilized as described previously. Blocking was performed using blocking solution (Sigma Aldrich, #DUO82007) for 1 h at 37 °C, followed by an overnight incubation at 4 °C with anti-PAD4 antibody combined with one of the following primary antibodies: anti-MYH9, anti-GCA, anti-MYL6, anti-NCF1, or anti-STOM, diluted to their optimized concentrations in antibody diluent (Sigma Aldrich, #DUO82008). After washing, cells were incubated with Duolink PLA probe anti-rabbit PLUS and probe anti-mouse MINUS for 1 h at 37 °C. The ligation reaction was performed for 30 min at 37 °C using the ligation solution, followed by amplification for 100 min at 37 °C using the amplification solution. Signals were detected and analyzed using an immunofluorescence microscope. Negative controls included staining with either anti-PAD4 alone or no primary antibody, while NCF1 combined with PAD4 was used as a positive control.

### ELISA for autoantibodies of patients with RA against citrullinated peptides

Myosin-9 peptides used were: LAAENRLTEME (R867), RQAQQERDELADE (R1703), EEEAQRANASR (R1888), and ADAMNREVSSL (R1912), in which the underlined R was either citrulline or Arg, and similarly for MYL6: YVEGLRVFDKE (R94) and MYL12A: TTMGDRFTDEE (R132). Peptides were coated at 200ng/well on 96-well ELISA plates in a 0.1M carbonate (pH 9.6) overnight, washed with phosphate-buffered saline with 0.05% Tween-20 (PBST), and blocked with 10% nonfat milk in PBST for 2 h. After washing with PBST four times, patient sera were diluted 1:500 in 10% nonfat milk and incubated for 2 h, plates washed, and incubated with 100 μl of 1:2000 diluted secondary antibody, HRP-conjugated goat anti-human IgG, in 10% nonfat milk for 45 min. TMB substrate was added for approximately 10 min. The color reaction was stopped with 0.5 N H_2_SO_4,_ and plates were read at 450 nm and calibrated at 630 nm using a plate reader. Reagents used were freshly prepared immediately before use and allowed to warm to room temperature. The absorbance values obtained with each unmodified peptide were subtracted from the values with the corresponding citrullinated peptide, and positive values were considered to indicate higher IgG binding of the citrullinated peptide (cit > R). Negative values mean lack of citrulline-selectivity (*i.e.*, higher IgG binding to the unmodified peptide) and were set to 0 for data analysis. Values < 0.05 were considered likely background.

### Statistics

For non-paired sample sets with non-Gaussian distribution, the Mann-Whitney U test was used. For paired sample sets, we used the Wilcoxon matched-pairs signed rank test. GraphPad Prism 10.4.1 was used for these analyses. Results were considered statistically significant at *p* < 0.05.

## Data availability

All data will be made available for research purposes upon request.

## Supporting information

This article contains supporting information.

## Conflict of interest

The authors declare that they do not have any conflicts of interest with the content of this article.
