# Supplementary material for: Association of protein arginine deiminase 4 with the myosin-9 motor complex
Source: J Biol Chem. 2025 Jul 22;301(9):110513. doi: 10.1016/j.jbc.2025.110513 (PMC12390935; doi:10.1016/j.jbc.2025.110513)

## Supplemental data:

**Fig. S1. Representative mass spectra of PAD4 and myosin-9 in the anti-PAD4 immunoprecipitates.**

**Fig. S2. Mass spectra of MYL12A, MYL6, MYL9, LCP1, STOM and FCN1 in the same immunoprecipitates.**

**Fig. S3. Two mass spectra each from DYSF, GCA, and IQGAP in the same immunoprecipitates.**

**Fig. S4. A. Additional co-immunoprecipitation experiment: anti-myosin-9 immunoblot of immunoprecipitates from THP-1 cells with two different anti-PAD4 antibodies (lane 1, rabbit polyclonal; lane 2, rabbit antibody from SinoBiological). Lane 3 is total lysates, lane 4 insoluble material, lane 6 anti-MYH9 immunoprecipitate. Upper panel is a shorter (6 sec) exposure; lower panel is a longer (53 sec) exposure.**

**Fig. S5. Four mass spectra of citrullinated peptides from myosin-9 and one each from MYL12A and MYL6 in the same immunoprecipitates.**

# Supplemental Fig. S1

## MS/MS of PAD4 peptides

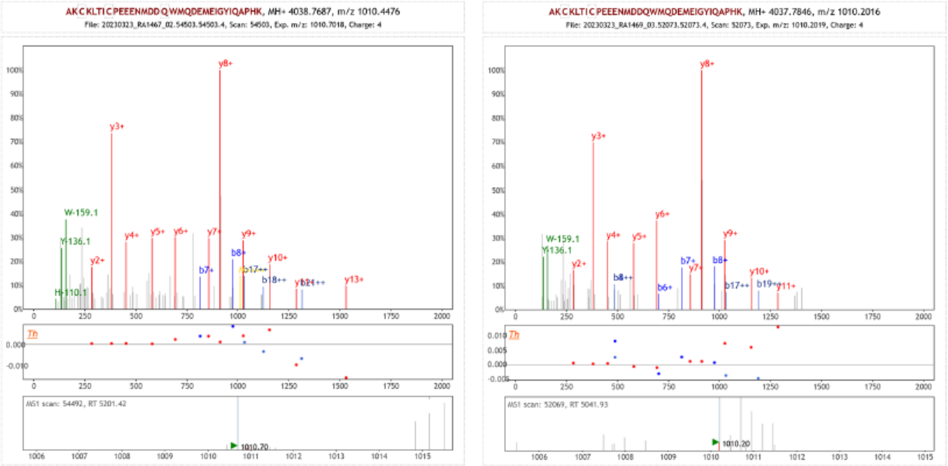


## MS/MS of MYH9 peptides

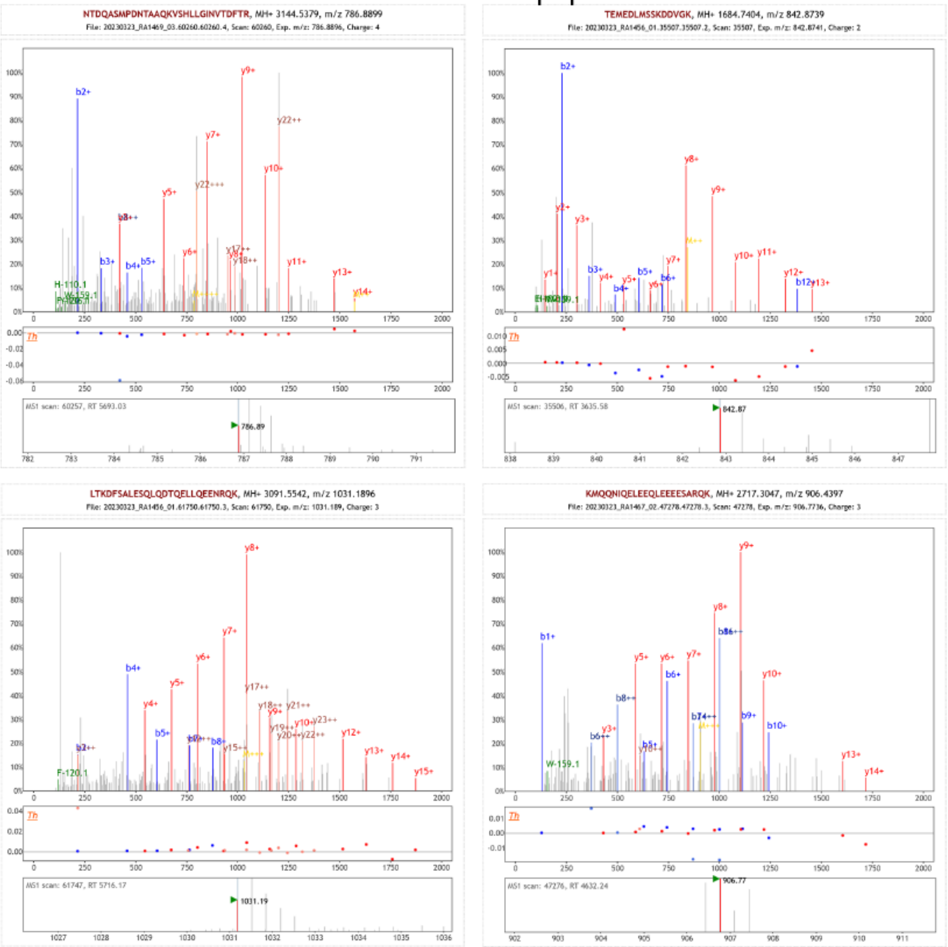

## Supplemental Fig. S2

MS/MS of MYL12A peptide

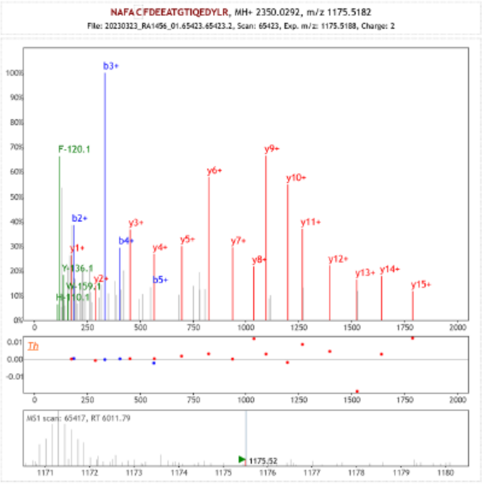

MS/MS of MYL6 peptide

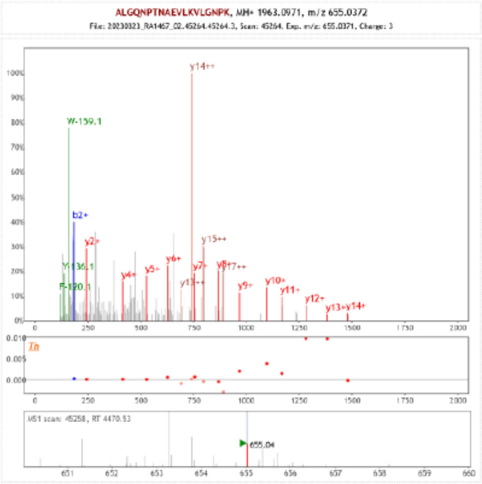

MS/MS of MYL9 peptide

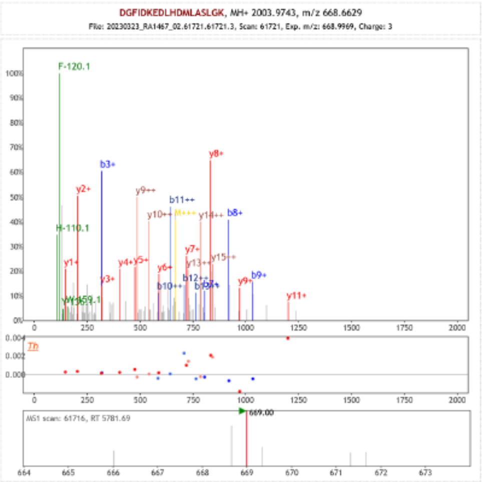

MS/MS of LCP1 peptide

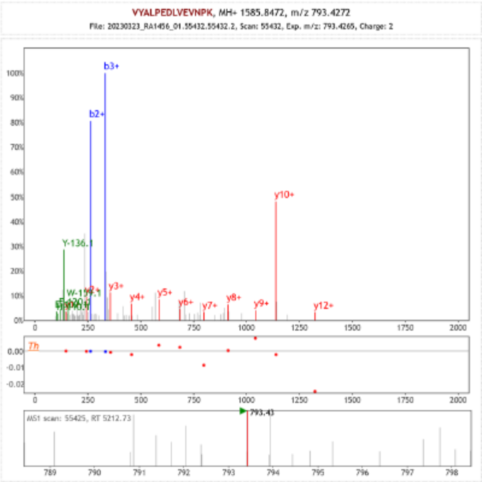

MS/MS of STOM peptide

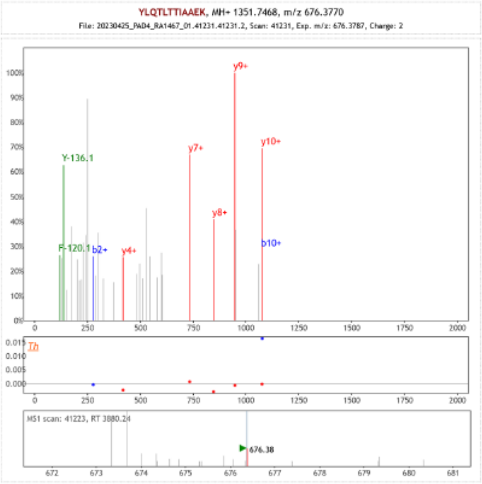

MS/MS of FCN1 peptide

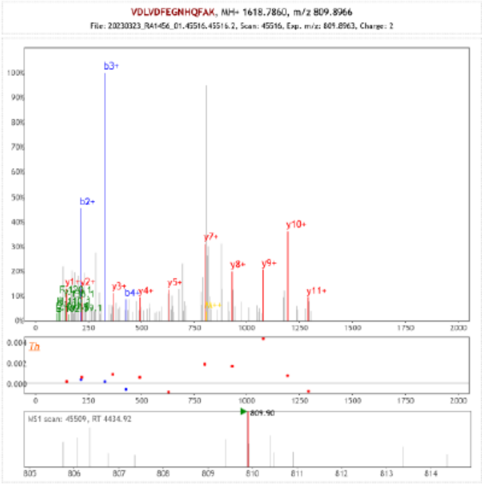

# Supplemental Fig. S3

MS/MS of DYSF peptides

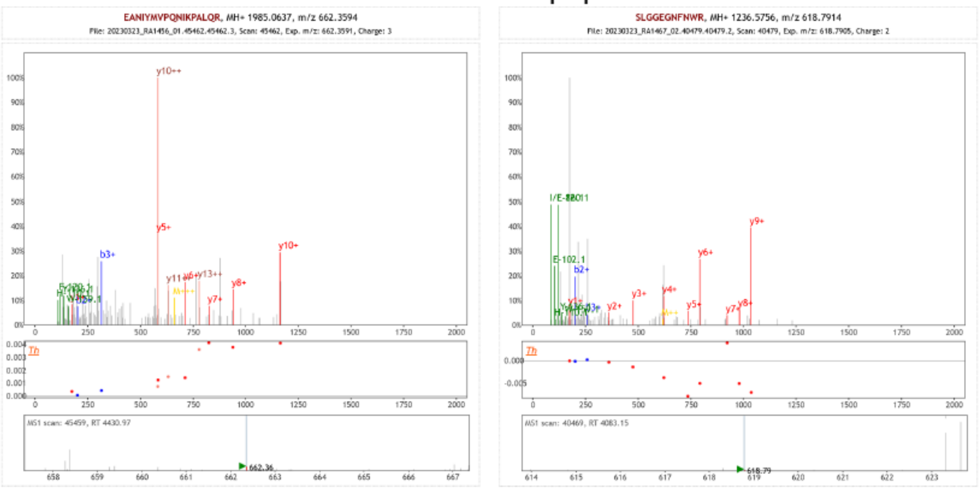


MS/MS of GCA peptides

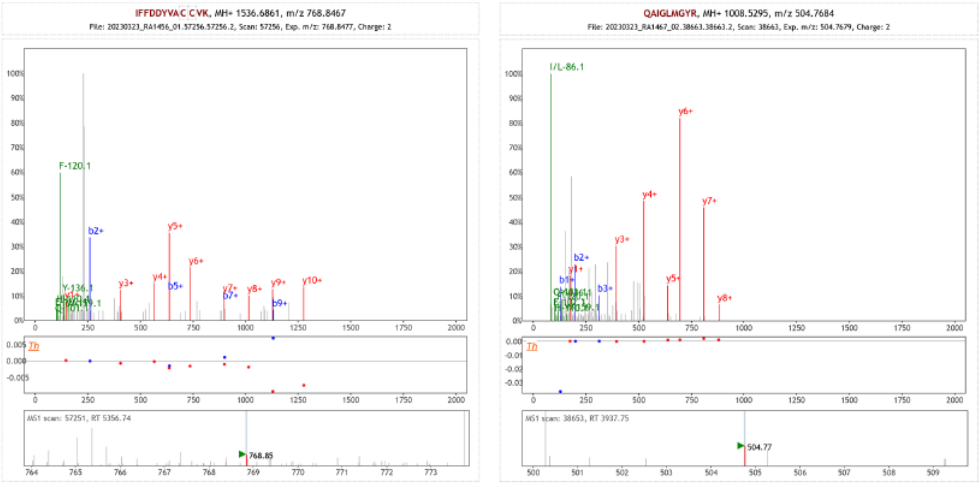


MS/MS of IQGAP peptides

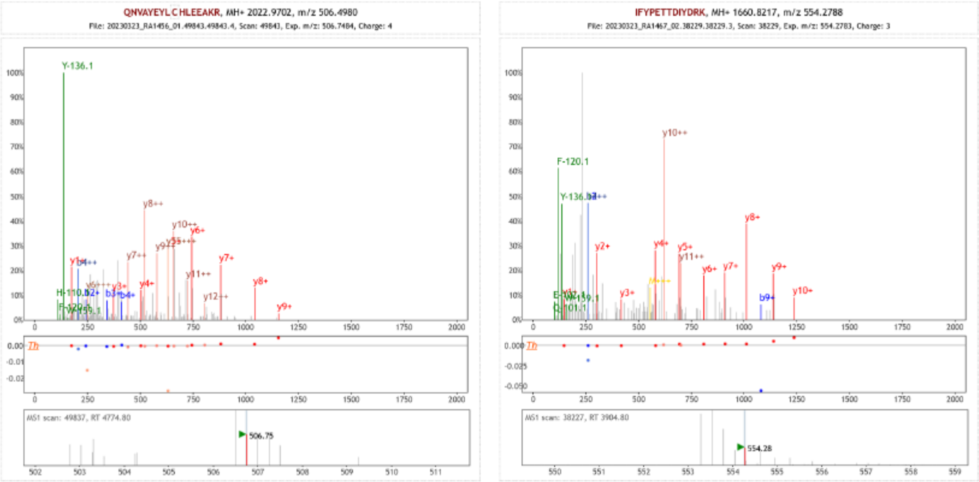

Suppl. Fig. S4

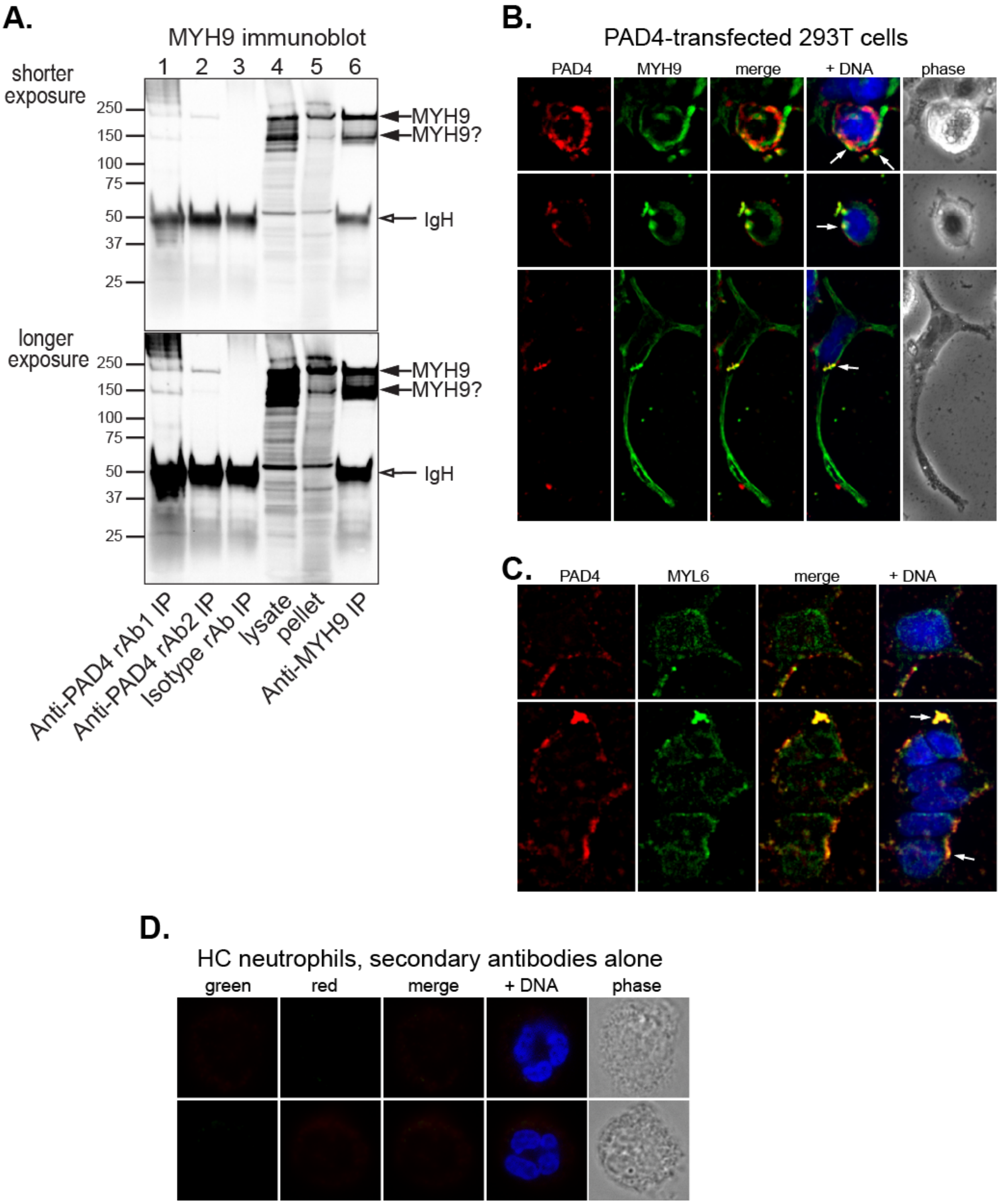
A.
MYH9 immunoblot
1 2 3 4 5 6
shorter exposure
longer exposure
250
150
100
75
50
37
25
MYH9
MYH9?
IgH
Anti-PAD4 rAb1 IP
Anti-PAD4 rAb2 IP
Isotype rAb IP
lysate
pellet
Anti-MYH9 IP
B.
PAD4-transfected 293T cells
PAD4
MYH9
merge
+ DNA
phase
C.
PAD4
MYL6
merge
+ DNA
D.
HC neutrophils, secondary antibodies alone
green
red
merge
+ DNA
phase

# Supplemental Fig. S5

MS/MS of citrullinated MYH9 peptides

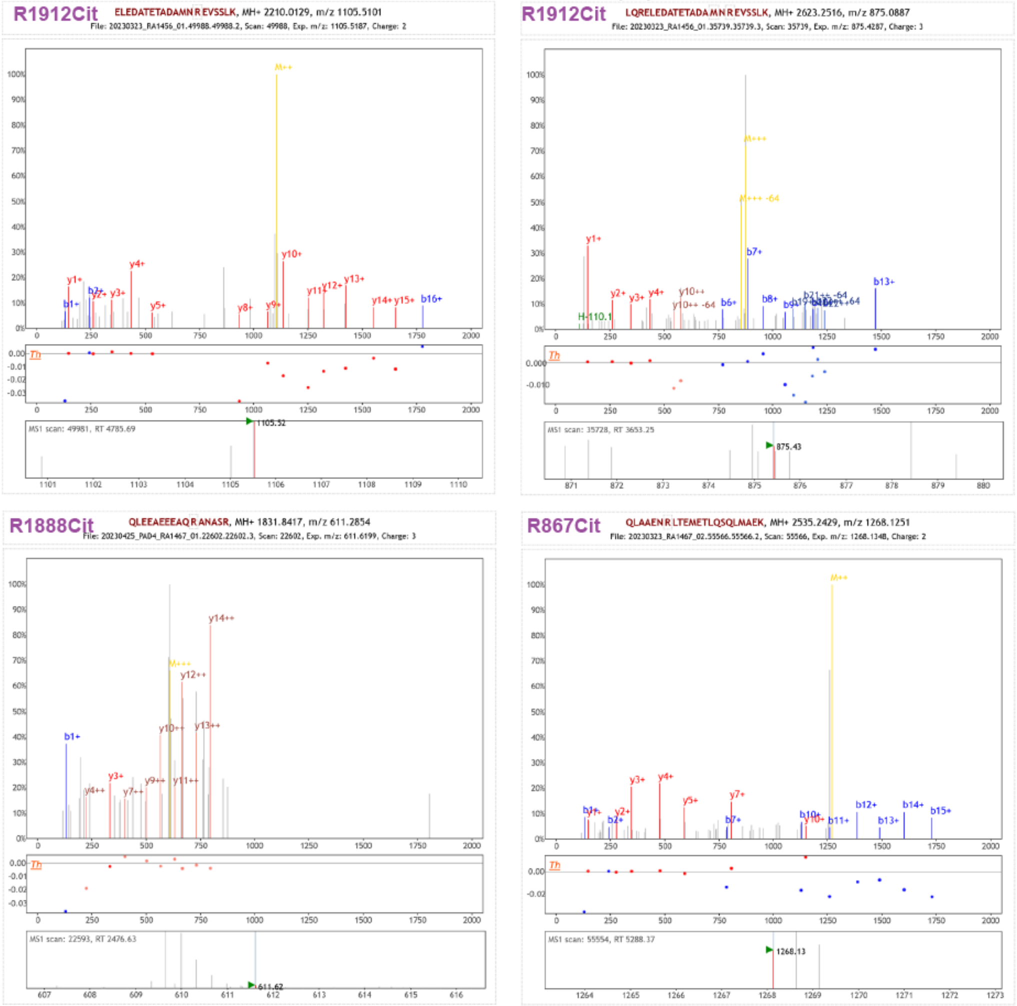


MS/MS of citrullinated MYL12A peptide

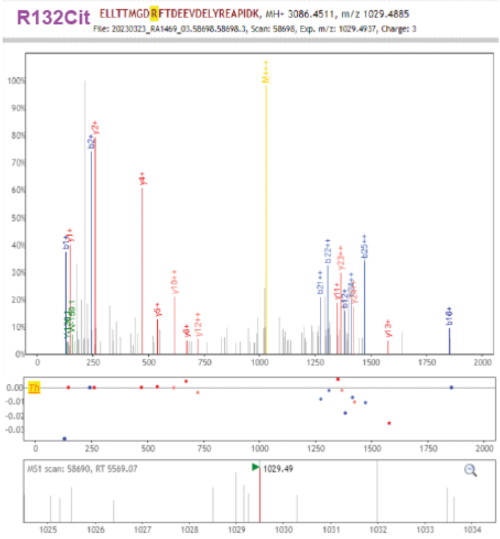


MS/MS of citrullinated MYL6 peptide

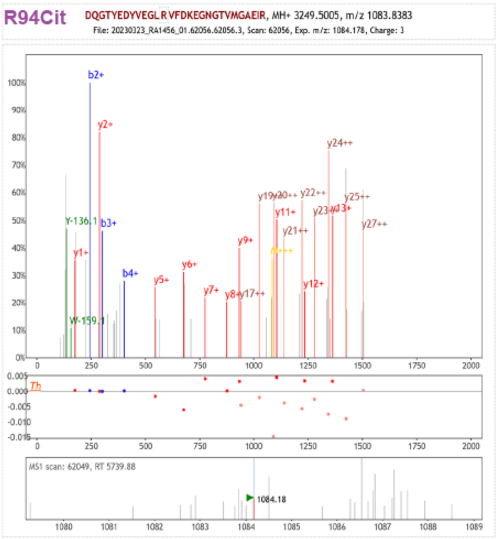

Supplement: Supplemental File [file mmc1.pdf]
